# Supplementary material for: Hot spot prediction in protein-protein interactions by an ensemble system
Source: BMC Syst Biol. 2018 Dec 31;12(Suppl 9):132. doi: 10.1186/s12918-018-0665-8 (PMC6311905; doi:10.1186/s12918-018-0665-8)
Supplement: Supplementary file 2 — All relevant MATLAB code. A simple Matlab implement of our predictor. (ZIP 19,742 kb) [file 12918_2018_665_MOESM2_ESM.zip › Additional file 2/Read ASA/ASA website/SKEMPI/1REWA.htm]

 NetSurfP - Protein Surface Accessibility and Secondary Structure Predictions

  

|  |  |  |
| --- | --- | --- |
|  |  | NetSurfP - Protein Surface Accessibility and Secondary Structure Predictions  Technical University of Denmark |

  


---

```
# For publication of results, please cite:
# A generic method for assignment of reliability scores applied to solvent accessibility predictions.
# Bent Petersen, Thomas Nordahl Petersen, Pernille Andersen, Morten Nielsen and Claus Lundegaard
# BMC Structural Biology 2009, 9:51 doi:10.1186/1472-6807-9-51
#
# Column 1: Class assignment - B for buried or E for Exposed - Threshold: 25% exposure, but not based on RSA
# Column 2: Amino acid
# Column 3: Sequence name
# Column 4: Amino acid number
# Column 5: Relative Surface Accessibility - RSA
# Column 6: Absolute Surface Accessibility
# Column 7: Z-fit score for RSA prediction
# Column 8: Probability for Alpha-Helix
# Column 9: Probability for Beta-strand
# Column 10: Probability for Coil
E S  1REWA                  1    0.776  90.947   0.152   0.003   0.003   0.994
E S  1REWA                  2    0.571  66.874   1.338   0.004   0.138   0.858
B C  1REWA                  3    0.077  10.797  -3.626   0.005   0.336   0.660
E K  1REWA                  4    0.391  80.449   1.013   0.003   0.718   0.279
E R  1REWA                  5    0.385  88.211  -0.090   0.002   0.816   0.182
E H  1REWA                  6    0.341  62.028   1.174   0.002   0.816   0.182
E P  1REWA                  7    0.514  72.937   0.882   0.001   0.900   0.099
B L  1REWA                  8    0.101  18.511   0.755   0.001   0.900   0.099
E Y  1REWA                  9    0.369  78.748   0.929   0.001   0.900   0.099
B V  1REWA                 10    0.031   4.749   0.267   0.001   0.900   0.099
E D  1REWA                 11    0.294  42.337   0.952   0.002   0.816   0.182
B F  1REWA                 12    0.068  13.688   0.491   0.023   0.655   0.322
E S  1REWA                 13    0.536  62.807   1.322   0.023   0.655   0.322
E D  1REWA                 14    0.485  69.946   0.373   0.022   0.552   0.426
B V  1REWA                 15    0.152  23.347   1.047   0.022   0.359   0.619
E G  1REWA                 16    0.429  33.754  -0.801   0.020   0.205   0.775
B W  1REWA                 17    0.239  57.600  -0.449   0.064   0.216   0.721
E N  1REWA                 18    0.494  72.263  -0.669   0.019   0.141   0.840
E D  1REWA                 19    0.360  51.934   0.069   0.021   0.279   0.699
B W  1REWA                 20    0.090  21.645   0.586   0.023   0.655   0.322
B I  1REWA                 21    0.043   7.881   0.373   0.021   0.756   0.223
B V  1REWA                 22    0.077  11.866   0.050   0.021   0.756   0.223
B A  1REWA                 23    0.153  16.894   0.814   0.021   0.451   0.528
B P  1REWA                 24    0.082  11.636  -0.047   0.019   0.141   0.840
E P  1REWA                 25    0.530  75.264   0.216   0.018   0.047   0.935
E G  1REWA                 26    0.393  30.905  -0.999   0.018   0.088   0.893
B Y  1REWA                 27    0.113  24.105   0.031   0.020   0.205   0.775
E H  1REWA                 28    0.347  63.137   0.287   0.021   0.451   0.528
B A  1REWA                 29    0.051   5.653  -0.572   0.022   0.552   0.426
B F  1REWA                 30    0.135  27.074  -0.808   0.021   0.756   0.223
B Y  1REWA                 31    0.237  50.711   0.225   0.021   0.756   0.223
B C  1REWA                 32    0.020   2.850  -0.406   0.023   0.655   0.322
E H  1REWA                 33    0.328  59.700  -0.084   0.021   0.451   0.528
B G  1REWA                 34    0.090   7.099  -2.200   0.021   0.451   0.528
E E  1REWA                 35    0.514  89.708   0.215   0.021   0.451   0.528
B C  1REWA                 36    0.078  10.993   0.168   0.022   0.359   0.619
E P  1REWA                 37    0.467  66.211  -1.222   0.064   0.216   0.721
B F  1REWA                 38    0.256  51.459  -0.197   0.052   0.084   0.864
B P  1REWA                 39    0.283  40.115  -0.818   0.053   0.043   0.903
B L  1REWA                 40    0.200  36.675  -0.151   0.113   0.043   0.844
E A  1REWA                 41    0.401  44.168  -1.679   0.115   0.016   0.868
E D  1REWA                 42    0.563  81.128  -1.818   0.058   0.017   0.925
E H  1REWA                 43    0.386  70.232  -1.467   0.053   0.043   0.903
B L  1REWA                 44    0.150  27.483  -0.825   0.053   0.043   0.903
E N  1REWA                 45    0.323  47.346  -0.882   0.018   0.047   0.935
B S  1REWA                 46    0.171  20.076  -2.245   0.058   0.017   0.925
B T  1REWA                 47    0.175  24.286  -1.581   0.257   0.016   0.727
B N  1REWA                 48    0.141  20.613  -1.117   0.522   0.016   0.462
B H  1REWA                 49    0.097  17.608  -1.065   0.802   0.014   0.185
B A  1REWA                 50    0.121  13.356  -1.412   0.911   0.033   0.057
B I  1REWA                 51    0.091  16.853   0.229   0.911   0.033   0.057
B V  1REWA                 52    0.020   3.013   0.923   0.918   0.063   0.019
B Q  1REWA                 53    0.115  20.593   0.643   0.918   0.063   0.019
B T  1REWA                 54    0.162  22.497  -0.674   0.911   0.033   0.057
B L  1REWA                 55    0.080  14.685   0.436   0.911   0.033   0.057
B V  1REWA                 56    0.082  12.557   0.334   0.879   0.010   0.111
E N  1REWA                 57    0.399  58.370   0.268   0.879   0.010   0.111
E S  1REWA                 58    0.394  46.235  -0.475   0.717   0.014   0.269
E V  1REWA                 59    0.351  53.995  -0.724   0.321   0.003   0.675
E N  1REWA                 60    0.437  63.904  -0.701   0.053   0.005   0.942
E S  1REWA                 61    0.506  59.338  -2.157   0.016   0.005   0.979
E K  1REWA                 62    0.754 155.098  -1.018   0.003   0.003   0.994
E I  1REWA                 63    0.314  58.053  -0.593   0.005   0.015   0.979
E P  1REWA                 64    0.438  62.081  -0.873   0.005   0.015   0.979
E K  1REWA                 65    0.446  91.783   0.031   0.005   0.015   0.979
B A  1REWA                 66    0.177  19.505  -0.819   0.005   0.045   0.951
B C  1REWA                 67    0.065   9.112  -0.218   0.021   0.279   0.699
B C  1REWA                 68    0.034   4.760  -0.649   0.022   0.552   0.426
B V  1REWA                 69    0.178  27.389   1.092   0.021   0.451   0.528
B P  1REWA                 70    0.118  16.773  -0.245   0.021   0.279   0.699
E T  1REWA                 71    0.348  48.240   0.220   0.021   0.279   0.699
E E  1REWA                 72    0.492  85.917   0.770   0.021   0.279   0.699
B L  1REWA                 73    0.203  37.114  -0.036   0.021   0.279   0.699
E S  1REWA                 74    0.468  54.791  -0.347   0.004   0.085   0.910
E A  1REWA                 75    0.342  37.732  -1.090   0.004   0.197   0.799
B I  1REWA                 76    0.065  12.099   0.373   0.004   0.616   0.381
B S  1REWA                 77    0.192  22.491  -0.021   0.002   0.816   0.182
B M  1REWA                 78    0.039   7.864   0.523   0.001   0.900   0.099
B L  1REWA                 79    0.047   8.642   0.587   0.001   0.959   0.040
B Y  1REWA                 80    0.089  19.126   0.405   0.001   0.959   0.040
B L  1REWA                 81    0.118  21.569   0.344   0.001   0.959   0.040
B D  1REWA                 82    0.207  29.800   0.527   0.003   0.718   0.279
E E  1REWA                 83    0.718 125.435   0.078   0.004   0.085   0.910
E N  1REWA                 84    0.770 112.699  -0.730   0.003   0.003   0.994
E E  1REWA                 85    0.460  80.310  -0.138   0.005   0.045   0.951
E K  1REWA                 86    0.389  80.079   0.770   0.004   0.616   0.381
B V  1REWA                 87    0.134  20.642   1.384   0.001   0.900   0.099
E V  1REWA                 88    0.309  47.478   1.284   0.001   0.959   0.040
B L  1REWA                 89    0.225  41.198   0.876   0.001   0.959   0.040
E K  1REWA                 90    0.346  71.131   1.321   0.001   0.959   0.040
E N  1REWA                 91    0.491  71.824   1.444   0.003   0.718   0.279
B Y  1REWA                 92    0.145  30.965   1.091   0.020   0.205   0.775
E Q  1REWA                 93    0.738 131.718   0.868   0.018   0.019   0.964
E D  1REWA                 94    0.715 102.974   0.823   0.018   0.019   0.964
B M  1REWA                 95    0.078  15.568   0.899   0.022   0.359   0.619
E V  1REWA                 96    0.316  48.569   1.700   0.018   0.846   0.136
B V  1REWA                 97    0.126  19.305   0.212   0.018   0.846   0.136
E E  1REWA                 98    0.521  90.984   1.083   0.021   0.756   0.223
E G  1REWA                 99    0.566  44.528   1.055   0.023   0.655   0.322
B C  1REWA                100    0.093  12.987  -0.764   0.022   0.359   0.619
E G  1REWA                101    0.423  33.251  -0.273   0.005   0.262   0.733
B C  1REWA                102    0.189  26.493  -1.580   0.004   0.138   0.858
E R  1REWA                103    0.684 156.682   0.621   0.005   0.015   0.979

Explain the output.  Go back.
```

---
